# Influence of unstable shoes on women with lumbopelvic postpartum pain: randomized clinical trial

**DOI:** 10.1590/1516-3180.2020.0738.R1.0402021

**Published:** 2021-06-11

**Authors:** Raquel Díaz-Meco Conde, Beatriz Ruiz Ruiz, Margarita Rubio Alonso, César Calvo-Lobo, Carmen de Labra, Daniel López-López, Carlos Romero Morales

**Affiliations:** I PT, MSc, PhD. Professor and Researcher, School of Sport Sciences, Universidad Europea de Madrid, Villaviciosa de Odón, Madrid, Spain.; II PT, MSc, PhD. Lecturer and Researcher, School of Sport Sciences, Universidad Europea de Madrid, Villaviciosa de Odón, Madrid, Spain.; III MD, PhD. Lecturer and Researcher, School of Biomedical and Health Sciences, Universidad Europea de Madrid, Villaviciosa de Odón, Madrid, Spain.; IV PT, MSc, PhD. Lecturer and Researcher, School of Nursing, Physiotherapy and Podiatry, Universidad Complutense de Madrid, Madrid, Spain.; V PhD. Lecturer and Researcher, NEUROCom Group, Department of Physiotherapy, Medicine and Biomedical Sciences, School of Nursing and Podiatry, Universidade da Coruña, Ferrol, Spain.; VI PhD, BSc, MSc, DPM. Senior Lecturer and Researcher, Health and Podiatry Group, Department of Health Sciences, School of Nursing and Podiatry, Universidade da Coruña, Ferrol, Spain.; VII PT, MSc, PhD. Senior Lecturer and Researcher, School of Sport Sciences, Universidad Europea de Madrid, Villaviciosa de Odón, Madrid, Spain.

**Keywords:** Postpartum period, Low back pain, Shoes, Pregnancy, Postpartum, Backache, Footwear, Lumbopelvic pain

## Abstract

**BACKGROUND:**

Back pain is a normal symptom during pregnancy and is expected to become worse beyond the first three months after childbirth.

**OBJECTIVES:**

To determine the effectiveness of wearing unstable shoes instead of conventional shoes, regarding pain intensity, low back mobility and stability, among women with lumbopelvic pain (LPP) during the postpartum period.

**DESIGN AND SETTING:**

Prospective, single-blinded, randomized clinical trial conducted at a podiatry and physiotherapy clinical center.

**METHODS:**

A nine-week program of wearing either unstable shoes (A) or conventional shoes (B) was implemented. The following outcomes were measured in three assessments: pain intensity, using a visual analogue scale (VAS); low-back mobility, using a modified Schober test; and stability, using a pressure platform.

**RESULTS:**

The lateral stability speed, anterior stability speed and anterior center of pressure (COP) showed significant (P < 0.05) decreases in the unstable shoes group after nine weeks, in relation to the conventional group. Intra-group measurements showed significant differences (P < 0.05) in VAS between the second and third assessments and between the first and third assessments in both groups. Intra-group evaluations also showed statistically significant differences (P < 0.05) in the lateral stability speed and anterior stability speed.

**CONCLUSIONS:**

Unstable shoes were effective in decreasing the pain intensity at five and nine weeks in women with postpartum LPP. In addition, their use produced decreases in lateral stability speed, anterior stability speed and anterior COP at nine weeks.

## INTRODUCTION

Back pain is a normal symptom during pregnancy and is expected to become worse beyond the first three months after childbirth.^1^ Several authors have indicated that from 8% to 20% of women present nonspecific lumbopelvic pain (LPP), two to three years after childbirth, which decreases their quality of life and interferes with their daily activities.^2^^,^^3^^,^^4^ Gutke et al.^5^ reported that LPP was related to lumbar instability due to the structural changes produced during the pregnancy.

Postpartum LPP can be assessed based on questionnaires and clinical examinations.^3^ In addition, Fritz et al.^6^ showed the importance of LPP classification for choosing the optimal intervention strategy. The clinical features of postpartum LPP have been reported to be pain, disability, lack of range of motion (ROM) in the sacroiliac joint, kinesiophobia, reduced quality of life and delayed resumption of doing exercise activities.^5^^,^^7^

Several authors have studied the influence of the core muscles on LPP. For example, Hodges et al.^8^ found that individuals with low back pain presented decreased transversus muscle activity. Moreover, Teyhen et al.^9^ reported that individuals with LPP showed reduction in the thickness of deep abdominal muscles. Exercise programs have been found to be effective in reducing the incidence of LPP, and also in decreasing the number of LPP symptoms, such pain and disability.^10^^,^^11^ Stuge et al.^12^ conducted a physical therapy program focused on specific stabilizing exercises for women with pelvic girdle pain and showed that these exercises produced benefits regarding pain, functionality and quality of life. 

Previous studies have found benefits with regard to increasing the muscle activity in different areas through using unstable shoes. Romkes et al.^13^ carried out a 3D gait analysis among healthy individuals with and without unstable shoes and reported that changes in movement patterns occurred in the group wearing unstable shoes, such as increased ankle dorsiflexion ROM and muscle activity. In addition, Nigg et al.^14^ reported that there was an increase in electromyography activity in the tibialis anterior in healthy subjects who were using unstable shoes. Regarding the effectiveness of unstable shoes in relation to the trunk muscles and back pain, Nigg et al.^15^ reported that unstable shoes may be used to improve low back pain symptoms in golfers, without adverse effects. Lison et al.^16^ performed a gait analysis on healthy participants wearing unstable shoes and showed that they presented increased erector spinae and rectus abdominis muscle activity. Moreover, in a comparative study between patients with chronic low back pain and healthy participants, unstable shoes were reported to have the effect of decreasing low back pain.^17^

Currently, there is a lack of randomized clinical trials (RCTs) regarding the effectiveness of unstable shoes, especially among women who suffer from LPP during the postpartum period. We hypothesized that women with LPP during this period could benefit from wearing unstable shoes. 

## OBJECTIVE

The primary aim of the present study was to determine the effectiveness of wearing unstable shoes instead of conventional shoes, regarding pain intensity, among women with LPP during the postpartum period. Therefore, as a secondary objective, the aim was to assess the effectiveness of wearing unstable shoes with regard to low back mobility and stability in this population. 

## METHODS

### Design

The present study was a prospective, single-blinded, randomized clinical trial (registered at ClinicalTrials.gov: NCT03065270) that was conducted between October 2013 and July 2014. It followed the guidelines of the Consolidated Standards of Reporting Trials (CONSORT).

### Participants

**Figure 1.**
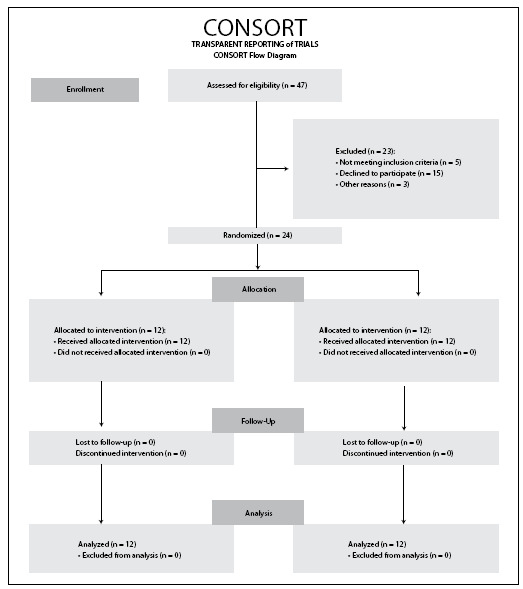
Flow chart describing the patient recruitment and assessment process.

Twenty-four women who had been diagnosed with LPP during the postpartum period were included. They were randomly divided into two groups (A and B): group A (n = 12) wearing unstable shoes; and group B (n = 12) wearing conventional shoes. The enrollment of patients was carried out by a specialist medical doctor with more than 15 years in the field of gynecology. All the patients were recruited at the Hospital Quirón, in Madrid, Spain. Figure 1 presents a flow chart describing the patient recruitment and assessment process.

The inclusion criteria for the study subjects were that they needed to be women aged from 18 to 40 years old, who were primiparous with LPP, had given birth 8 to 12 weeks previously, presented visual analogue scale (VAS) scores of at least 3 out of 10 points and were capable of walking autonomously.^17^ The following occurrences were exclusion criteria: implementation of physical therapy interventions, body mass index (BMI) higher than 30 kg/m^2^, lower limb injury within the last year, fractures, hemorrhage, induced pregnancy,^18^ systemic disease, infections, vaginal prolapses, shoe size smaller than 35 or larger than 42 (European sizes), dizziness or balance disorders.^17^

The sample size was determined to be a convenience sample of 24 subjects, based on data from a previous study.^18^

### Ethics

The Research Ethics Committee of the Hospital Universitario de Getafe (Madrid, Spain) approved the study (under protocol no. UEM-DOL-2011-01; dated September 28, 2011). The Declaration of Helsinki was respected throughout the study and a consent statement was signed by all the participants before their inclusion in the study.

### Procedure

Prior to the intervention, the subjects performed a short program of dynamic exercises to familiarize themselves with the shoes that they would be using. As recommended by Stewart et al.,^19^ the assessments were started only if all the women were accustomed to the shoes and were able to walk comfortably.

**Figure 2.**
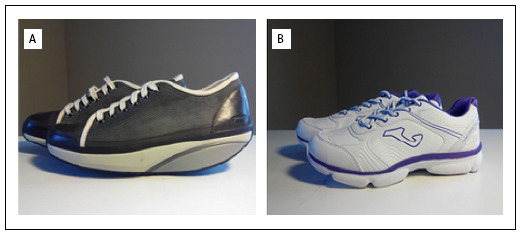
Unstable shoes (A) and conventional shoes (B).

In the present study, unstable shoes were assigned to the A group (Masai Barefoot Technology, Masai Marketing and Trading AG, Winterthur, Switzerland) (Figure 2A) and conventional shoes were assigned to the B group (Joma, Portillo de Toledo, Spain) (Figure 2B). Both groups performed a nine-week program in which the following recommendations were made: the subjects wore their shoes for one hour per day starting on the first day; on the third day, they increased the duration of the intervention to three hours per day; and on the fifth day, they reached four hours of intervention per day. Through this gradual increase in utilization, the patients did not suffer any problems regarding adaptation to the footwear. From the fifth day to the end of the intervention at nine weeks, all the patients wore the shoes for four hours per day.

### Randomization

The participants were randomized into an unstable shoes group or a conventional shoes group by means of the free software randomized.org, with a 1:1 ratio. Individuals were informed not to discuss the randomization groups with the outcome measurements evaluator.

### Outcome measurements

All measurements were performed by a blinded examiner who did not know the group to which the individuals had been assigned. For this study, a total of three assessments were carried out: at baseline, five weeks and nine weeks. 

Firstly, pain intensity was evaluated using a VAS of 10 cm, ranging from 0 (no pain) to 10 cm (the worst pain imaginable). The subjects marked their pain intensity on the scale using a marker pen. In a previous study, the VAS was considered to be a reliable and valid tool for evaluating pain intensity caused by mechanical stimulation.^20^

Secondly, low back mobility was assessed by means of a modified Schobert test, following the guidelines of Tousignant et al.^21^ The subjects were placed in a standing position and the evaluator marked out the midline of the lumbar spine at the posterosuperior iliac spine (lower landmark) at the level of L4/L5, using a pen. The evaluator also marked out a second line at a distance of 15 cm from the original one (higher landmark). Then the patient performed active anterior trunk flexion, without reaching pain, under the following order: “keep your knees straight and bend forward to touch your toes”. The new distance between the lower and higher landmarks was measured and the subject then returned to the neutral position. The difference in the distance between the skin marks initially made in the neutral position and the new marks made in the trunk flexion position was used to indicate the quantity of flexion.^21^ After each assessment, the marks were removed from the skin using alcohol. 

Lastly, center of pressure (COP) measurements to assess stability were made using a floor-mounted capacitance transducer platform (Medicapteurs, Balma, France). The patients were placed in a standing position with their feet at the width of their pelvis. They were told to stay in a comfortable position, looking straight ahead, with their eyes open during the 30 seconds that the test lasted. The mean of three 30-second tests done in quick succession was recorded for each measurement.^22^ This was followed by a five-minute resting period and then another set of tests. The following variables were recorded from the transducer platform: the velocities of the center of gravity developed in the frontal and sagittal planes (lateral and anterior stability speed variables, respectively); the mobility of the center of gravity in the frontal and sagittal planes (lateral and anterior COP mobility variables, respectively).

### Statistical analysis

The data analysis was performed using the SPSS package for Windows (version 23.0, IBM Corp., Armonk, New York, United States). Firstly, the Shapiro-Wilk test was used to assess the normality assumption. Secondly, a comparative analysis between groups was performed. For parametric data analysis, Student’s t test was used and for nonparametric data analysis, the Mann-Whitney U test was used. Lastly, for intra-group comparisons, the Wilcoxon rank test was performed. For all statistical tests, an α error of 0.05 (95% confidence interval) and a desired power of 90% (β error of 0.1) were used.

## RESULTS

The sociodemographic data did not show any statistically significant differences (P > 0.05) (Table 1). The lateral stability speed, anterior stability speed and anterior center of pressure (COP) were significantly lower (P < 0.05) in the unstable shoes group after the nine weeks of the intervention (Table 2). The intra-group measurements (Table 3) showed significant (P < 0.05) differences in VAS between the second and third assessments and between the first and third assessments, in both groups. The intra-group evaluations also found statistically significant differences (P < 0.05) in the variables of lateral stability speed and anterior stability speed.

**Table 1.** Sociodemographic data

Data	Unstable shoes	Control	P-value
Age, years	35 ± 4	34 ± 3	0.44
Body mass index, kg/m^2^	21.82 ± 2.56	22.65 ± 3.17	0.67
Weight increase during pregnancy, kg	12 ± 4	12 ± 5	0.84
Newborn weight, kg	3.11 ± 0.30	2.96 ± 0.40	0.44

Values are mean ± standard deviation unless otherwise indicated.

**Table 2.** Comparison measurements between intervention groups

Measurement	Unstable shoes (n = 12)	Control (n = 12)	P-value
**Visual analogue scale**
Baseline	6.17 ± 0.34	5.75 ± 1.54	0.55
Five weeks	4.33 ± 2.23	5.33 ± 2.10	0.27
Nine weeks	2.42 ± 2.54	4.33 ± 2.53	0.11
**Lumbar mobility**
Baseline	6.80 ± 1.38	6.52 ± 1.22	0.48
Five weeks	6.67 ± 1.88	6.75 ± 1.89	0.82
Nine weeks	6.72 ± 2.18	6.75 ± 1.46	0.93
**Lateral stability speed**
Baseline	1.88 ± 0.34	1.81 ± 0.54	0.50
Five weeks	1.41 ± 0.35	1.71 ± 0.44	0.09
Nine weeks	1.28 ± 0.30	1.72 ± 0.40	0.01^*^
**Anterior stability speed**
Baseline	2.20 ± 0.70	2.07 ± 0.95	0.46
Five weeks	1.56 ± 0.46	1.95 ± 0.62	0.23
Nine weeks	1.43 ± 0.44	1.98 ± 0.55	0.03^*^
**Lateral center of pressure mobility**
Baseline	1.86 ± 0.64	1.57 ± 0.69	0.42
Five weeks	1.33 ± 0.65	1.48 ± 0.57	0.46
Nine weeks	1.24 ± 0.45	1.45 ± 0.46	0.25
**Anterior center of pressure mobility**
Baseline	2.70 ± 1.20	2.80 ± 1.57	0.42
Five weeks	2.13 ± 0.83	2.34 ± 1.29	0.81
Nine weeks	1.67 ± 0.80	2.66 ± 1.34	0.04^*^

Values are mean ± SD unless otherwise indicated; ^*^P-value showing statistically significant difference.

**Table 3.** Comparison of intra-group measurements between the intervention group (unstable shoes) and control group (conventional shoes)

Comparison	Unstable shoes P-value (n = 12)	Control P-value (n = 12)
**Visual analogue scale**
Baseline - five weeks	0.07	0.34
Five weeks - nine weeks	0.00^*^	0.03^*^
Baseline - nine weeks	0.00^*^	0.03^*^
**Lumbar mobility**
Baseline - five weeks	0.08	0.93
Five weeks - nine weeks	0.39	0.72
Baseline - nine weeks	0.47	0.62
**Lateral stability speed**	0.01^*^	0.98
**Anterior stability speed**	0.01^*^	0.86
**Lateral center of pressure mobility**	0.15	0.86
**Anterior center of pressure mobility**	0.06	0.21

^*^P-value showing statistically significant difference.

## DISCUSSION

To our knowledge, this was the first study to observe the effectiveness of unstable shoes among postpartum women. All the participants included in the present study presented LPP and showed decreases in pain intensity in the intervention at five and nine weeks. However, no significant differences were found between the groups with regard to VAS. Like in our study, Vieria et al.^23^ reported that there was a significant decrease in lumbar pain in subjects who used unstable shoes, in comparison with a control group, over a six-week follow-up period. In addition, Hodges and Mosley^24^ argued that altered postural motor control of the core muscles was related to pain episodes in which modified postural patterns were developed. Along the same lines, Nigg et al.^25^ showed that there was a low back pain reduction of 1.75/10 points in VAS after six weeks of using unstable shoes. Lisón et al.^16^ showed that there was a significant increase in electromyographic activity in the rectus abdominis and erector spinae muscles during gait, among subjects using unstable shoes. In addition, from those findings, these authors suggested that use of unstable shoes could be a potential intervention for strengthening trunk muscles and improving low back pain. 

Based on our data, no statistically significant differences were found in either group, regarding lumbar mobility. Armand et al.^17^ explained that increased lumbar lordosis and co-contraction of the trunk muscles in patients using unstable shoes could constitute an inhibitory mechanism against low back pain. Consequently, use of unstable shoes could have potential implications regarding lumbar spine ROM both in healthy and in low-back-pain populations.^16^^,^^26^

Our findings showed that there was a significant decrease in imbalance in the sagittal and frontal planes in the intervention group (unstable shoes). The values were more conclusive regarding stability speed and anterior mobility at nine weeks, between the groups. In addition, our findings suggested that changes to lower-limb biomechanics in postpartum women, produced through training on unstable surfaces, had benefits regarding imbalance of the COP. Ruhe et al.^27^ showed in a systematic review that imbalance of the COP was related to subjects who suffered low back pain. Thus, use of unstable shoes for improving COP imbalances could be a new interventional approach for patients with lumbar disorders. Moreover, in several studies, use of unstable shoes not only showed benefits for balance, but also showed benefits with regard to enhancement of shock absorption of ground reaction forces.^25^^,^^28^^,^^29^

The current study suggested that use of unstable shoes had benefits regarding pain intensity and improvement of COP imbalances, while being a relatively inexpensive and portable intervention. Treatment was implemented while the patients were performing other activities, such work or activities of daily life. In addition, unstable shoes training should be carried out within a physical therapy protocol. 

Several limitations were observed in this study. Firstly, no straight-leg-raise test was performed in this study, although this might have been useful for evaluating lower-limb and low-back disturbances. Secondly, the effects of wearing unstable shoes before childbirth have not yet been studied. It may be of interest to observe the effectiveness of use of unstable shoes by pregnant women.

## CONCLUSIONS

Use of unstable shoes was effective for decreasing pain intensity at five and nine weeks among women with postpartum LPP. In addition, their use produced decreases in lateral stability speed, anterior stability speed and anterior COP at nine weeks.
